# Sex differences and age-related changes in vertebral body volume and volumetric bone mineral density at the thoracolumbar spine using opportunistic QCT

**DOI:** 10.3389/fendo.2024.1352048

**Published:** 2024-02-15

**Authors:** Sebastian Rühling, Jonas Dittmann, Tobias Müller, Malek El Husseini, Jannis Bodden, Moritz R. Hernandez Petzsche, Maximilian T. Löffler, Nico Sollmann, Thomas Baum, Vanadin Seifert-Klauss, Maria Wostrack, Claus Zimmer, Jan S. Kirschke

**Affiliations:** ^1^ Department of Diagnostic and Interventional Neuroradiology, TUM School of Medicine and Health, Klinikum rechts der Isar, Technical University of Munich, Munich, Germany; ^2^ Department of Informatics, TUM School of Computation, Information and Technology, Technical University of Munich, Munich, Germany; ^3^ Department of Diagnostic and Interventional Radiology, University Medical Center Freiburg, Freiburg im Breisgau, Germany; ^4^ TUM-Neuroimaging Center, Klinikum rechts der Isar, Technical University of Munich, Munich, Germany; ^5^ Department of Diagnostic and Interventional Radiology, University Hospital Ulm, Ulm, Germany; ^6^ Department of Gynaecology, Interdisciplinary Osteoporosis Center, TUM School of Medicine and Health, Technical University of Munich, Munich, Germany; ^7^ Department of Neurosurgery, TUM School of Medicine and Health, Klinikum rechts der Isar, Technical University of Munich, Munich, Germany

**Keywords:** osteoporosis, opportunistic QCT, sex-differences, aging, menopause, bone geometry, vertebral fractures

## Abstract

**Objectives:**

To quantitatively investigate the age- and sex-related longitudinal changes in trabecular volumetric bone mineral density (vBMD) and vertebral body volume at the thoracolumbar spine in adults.

**Methods:**

We retrospectively included 168 adults (mean age 58.7 ± 9.8 years, 51 women) who received ≥7 MDCT scans over a period of ≥6.5 years (mean follow-up 9.0 ± 2.1 years) for clinical reasons. Level-wise vBMD and vertebral body volume were extracted from 22720 thoracolumbar vertebrae using a convolutional neural network (CNN)-based framework with asynchronous calibration and correction of the contrast media phase. Human readers conducted semiquantitative assessment of fracture status and bony degenerations.

**Results:**

In the 40-60 years age group, women had a significantly higher trabecular vBMD than men at all thoracolumbar levels (p<0.05 to p<0.001). Conversely, men, on average, had larger vertebrae with lower vBMD. This sex difference in vBMD did not persist in the 60-80 years age group. While the lumbar (T12-L5) vBMD slopes in women only showed a non-significant trend of accelerated decline with age, vertebrae T1-11 displayed a distinct pattern, with women demonstrating a significantly accelerated decline compared to men (p<0.01 to p<0.0001). Between baseline and last follow-up examinations, the vertebral body volume slightly increased in women (T1-12: 1.1 ± 1.0 cm^3^; L1-5: 1.0 ± 1.4 cm^3^) and men (T1-12: 1.2 ± 1.3 cm^3^; L1-5: 1.5 ± 1.6 cm^3^). After excluding vertebrae with bony degenerations, the residual increase was only small in women (T1-12: 0.6 ± 0.6 cm^3^; L1-5: 0.7 ± 0.7 cm^3^) and men (T1-12: 0.7 ± 0.6 cm^3^; L1-5: 1.2 ± 0.8 cm^3^). In non-degenerated vertebrae, the mean change in volume was <5% of the respective vertebral body volumes.

**Conclusion:**

Sex differences in thoracolumbar vBMD were apparent before menopause, and disappeared after menopause, likely attributable to an accelerated and more profound vBMD decline in women at the thoracic spine. In patients without advanced spine degeneration, the overall volumetric changes in the vertebral body appeared subtle.

## 1 Introduction

Osteoporosis is a systemic skeletal disorder characterized by compromised bone strength and an increased susceptibility to fragility fractures, most commonly affecting the vertebral column (1, 2). In this context, the thoracolumbar spine plays a crucial role in load-bearing and overall skeletal integrity, with the majority of factures occurring in this region (3). Variations in volumetric bone mineral density (vBMD), one of the major risk factors for fractures, and the relationship to bone geometry (e.g., vertebral body volume) are important aspects for understanding the age-related changes contributing to fragility fractures along the thoracolumbar spine (4).

Investigations into age- and sex-related alterations in vBMD have predominantly focused on the lumbar spine (L1-3) through population-based and opposite-sex twin studies (5–7). These studies have highlighted notable differences between women and men, particularly with women exhibiting higher lumbar vBMD, especially before menopause (5, 6). A recent study indirectly validated these findings using Hounsfield unit (HU) measurements (8). However, less is known about age- and sex-related vBMD patterns throughout the entire thoracolumbar spine, partly due to the challenges associated with labor-intensive and oftentimes manual multilevel assessments that are required to derive vBMD values. Moreover, few studies have addressed the volumetric changes using three-dimensional (3D) segmentation techniques to accurately measure true volume, as opposed to interpolation methods employing various geometric formulas on two-dimensional measurements (9, 10). A recent investigation into segmentation-based vertebral volumes found an age-related volume increase in the anterior lumbar column in men, with no significant changes in women (9).

Recent advantages in machine learning now allow (1) for non-invasive measurements of 3D bone geometry and vBMD, and (2) to study larger spinal sections with reduced effort (11–13). In the present study, opportunistic quantitative computed tomography (QCT) and deep learning-based image analysis software were employed that allowed the automated extraction of vBMD and vertebral body volume in a multiple follow-up setting. The study data was then utilized to thoroughly explore vBMD and vertebral body volume, aiming to (1) determine the age-related changes in vBMD and vertebral body volume variations at various stages of adulthood, (2) assess these age-related changes between sexes, and (3) investigate the relationship between vBMD and vertebral body volume.

## 2 Materials and methods

### 2.1 Study population

This retrospective study received approval from the local institutional review board, which waived the requirement for written informed consent. We conducted a retrospective search in our digital picture archiving and communication system (Sectra AB, IDS 7; Linkoeping, Sweden). We included all patients over the age of 40 years that received ≥7 contrast-enhanced or non-contrast multi-detector CT (MDCT) scans of the thoracolumbar spine over a period of ≥6.5 years between January 2006 and December 2021 (mean follow-up 9.0 ± 2.1 years).

Exclusion criteria comprised (1) metal implants, (2) insufficient image quality (e.g., due to artifacts), and (3) reported history of spine metastases or oncological diseases affecting the spine (including multiple myeloma). Eight additional patients were excluded during the analysis due to (1) later-detected osteolytic or osteoblastic lesions (n=4), (2) severe bony lesions adjacent to cortical bone (n=3), and (3) inflammatory changes (n=1) (**Supplementary Figure 1**). The final dataset included 168 adults (51 women, mean age at baseline 58.7 ± 9.8 years), encompassing 1554 MDCT scans and a total of 22720 vertebrae. The baseline diagnoses included solid tumors (e.g., gastrointestinal stromal tumor) (n=130), lymphoma (n=17), vascular disease (e.g., abdominal aortic aneurysm) (n=17), inflammation (n=2), and others (2).

### 2.2 Image acquisition

Scans were obtained using 20 different MDCT scanners from 4 different vendors (Philips Healthcare, Siemens Healthineers, GE Medical Systems, Toshiba Medical Systems). Depending on the clinical indication, some scans were performed after administration of an intravenous contrast medium (n=1480, Iomeron 400, Bracco), with imaging acquired in the arterial (n=202) or portal-venous phase (n=1278). All scans were acquired in helical mode with a peak tube voltage ranging from 80 kVp to 140 kVp, axial slice thickness of 0.9-5 mm, and adaptive tube load.

### 2.3 Evaluation of vertebrae

Three neuroradiologists (T.M., S.R., J.K.) screened all CT scans for vertebral fractures (VFs) and degenerative changes. Semiquantitative screening was performed on sagittal and coronal reformations using an open-source visualization and grading tool (https://github.com/malekosh/FX-Grader). Fractures were manually graded according to Genant et al. (14), with vertebrae classified as non-fractured (grade 0) or fractured according to height loss (grade 1, 20–25%; grade 2, 25%–40%; and grade 3, ≥40%). Abnormal morphometry related to developmental changes, like in Scheuermann disease or in degenerative spondyloarthropathy, were not rated as fractures. Vertebrae that had a fracture grade ≥1 were excluded from further vBMD and volumetric assessment at the corresponding level. Simultaneously, all vertebrae were manually assessed for bony degenerative changes and other abnormalities, which were categorized into (1) Schmorl’s herniation (upper or lower endplate), (2) non-osteoporotic fracture (e.g., malignant or traumatic), (3) osteolytic/osteoblastic bone metastasis without fracture, (4) severe sclerotic changes of the endplates or trabecular compartment, (5) lesions adjacent to cortical bone (e.g., osteophytes), and (6) unclear lesions. For volumetric evaluation, vertebrae falling into categories (1), (4), and (5) were classified as degenerated. Vertebrae falling into categories (2), (3), and (6) were excluded from any further volumetric and vBMD assessment. All other vertebrae were categorized into the non-degenerated group.

### 2.4 Data processing and scan-specific calibrations

**Figure 1:**
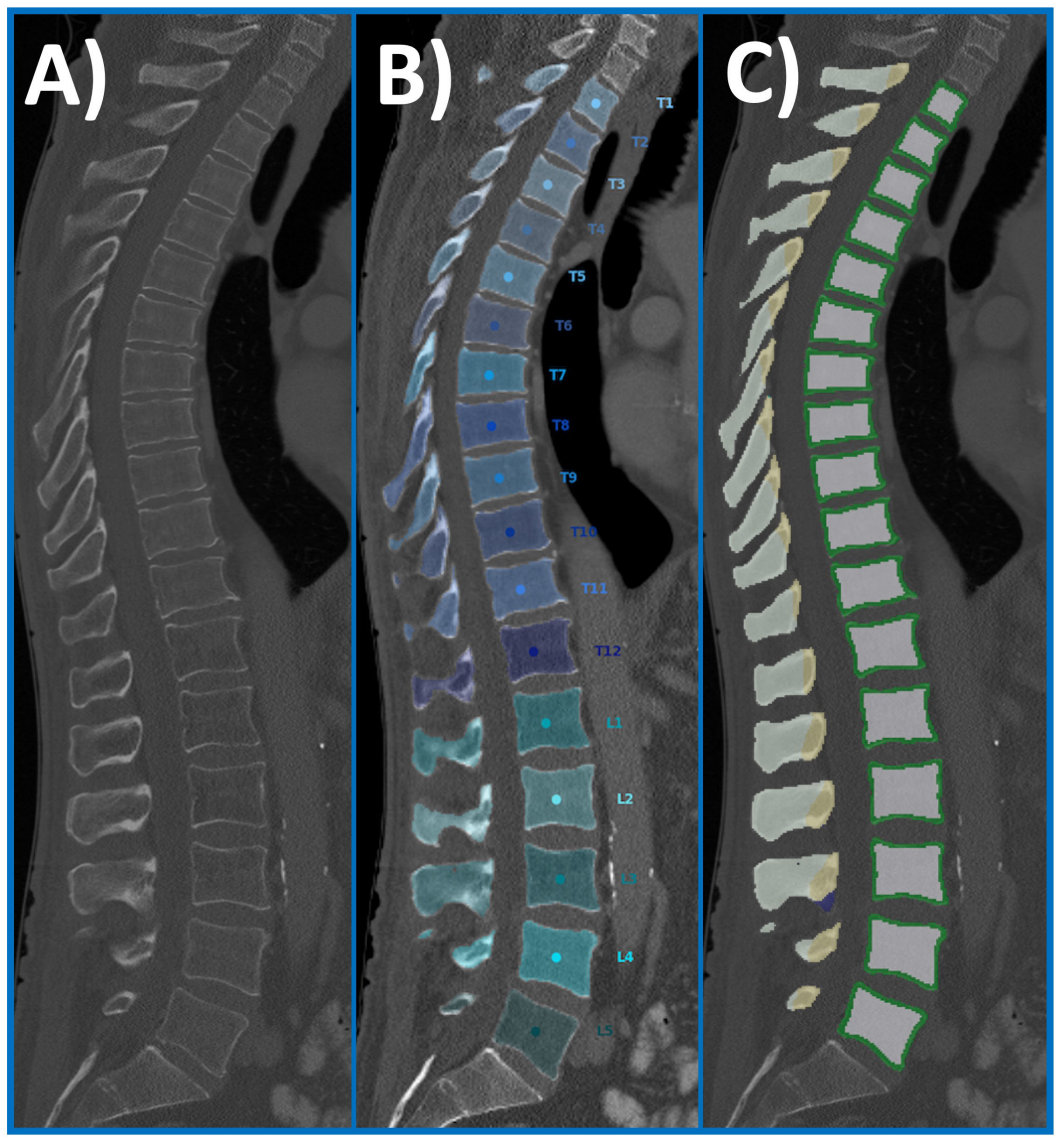
Overview of the automated spine processing pipeline. Exemplary sagittal CT scan of a 67-year-old man that serves as an input scan **(A)**. Labelling and automated segmentation of vertebral bodies (T1-L5) was achieved using a CNN-based framework **(B)**. The initial segmentation was used to create subregion masks of the vertebral body: masks of the cortical bone (green) and masks of the trabecular compartment (light grey). CNN, convolutional neural network; CT, computed tomography.

The CT data, including Digital Imaging and Communications in Medicine (DICOM) metadata, were exported, and converted into Neuroimaging Informatics Technology Initiative (NIfTI) format. Spine detection, vertebral labelling, and segmentations were performed using a convolutional neural network (CNN)-based framework (**Figures 1A, B**). Two masks were created for segmentation of the vertebral body: (1) masks of the cortical bone (**Figure 1C**, green) and (2) masks of the trabecular compartment (**Figure 1C**, grey). The sum of both masks was defined as the vertebral body volume (in cm^3^), and volumes were subsequently extracted for both degenerated and non-degenerated vertebrae.

The HU values were extracted from the trabecular segmentation masks, and tube voltage (in kVp) and scanner type were retrieved from DICOM metadata. For HU-to-vBMD conversion equations, kVp and scanner specific equations were used (vBMD=calibration factor x HU mg/cm^3^) (13). The calibration factors were previously determined by asynchronous phantom measurements with dedicated reference phantoms, resulting in scanner-specific (n=930) or generic vendor calibration factors (n=624). To minimize the contrast media-induced bias, the contrast media phase was automatically detected and subsequently corrected by linear regression for the respective contrast media phase, using the CNN-based framework (15). Quality control, including assessment of segmentation, vertebral body labelling, and contrast media phase correction, was jointly performed by two neuroradiologists (J.K., S.R.). Level-wise trabecular vBMD values were extracted from non-fractured and non-degenerated vertebrae (T1-L5). Additionally, extracted vBMD values at the lumbar spine were averaged over L1-L3 to classify patients into diagnostic categories (i.e., osteoporosis, osteopenia, normal vBMD) (16). If these vertebrae were not measurable, the vBMD values of the non-fractured and non-degenerated vertebrae of L4 and L5 were averaged.

### 2.5 Statistics

Statistical analyses were performed using Prism 9 (version 9.5.0, GraphPad Software), and p-values <0.05 were considered statistically significant. Standard descriptive statistics were calculated for the study set. The relationship between vBMD of each thoracic vertebra with the lumbar region (averaged values from L1 to L3 or L4 and L5) was determined using Pearson’s correlation coefficients. First, all fractured and degenerated vertebrae were excluded from the analysis. The calculation was then repeated a second time, including all fractured and degenerated vertebrae. The vBMD and volumetric analyses were computed separately for women and men in distinct age groups (i.e., 40-60 years and 60-80 years), and an unpaired t-test was used to assess differences in level-wise means. Wilcoxon test was performed to test whether mean longitudinal changes in vertebral body volume were different from zero. Pairs matched by age and sex were formed to study age-related changes in vBMD, and simple linear regression models were applied to compare the sexes. Using the baseline vertebral body volumes as a reference, we computed the Δ volume change up to the most recent examination.

## 3 Results

### 3.1 Osteoporotic fractures

**Figure 2:**
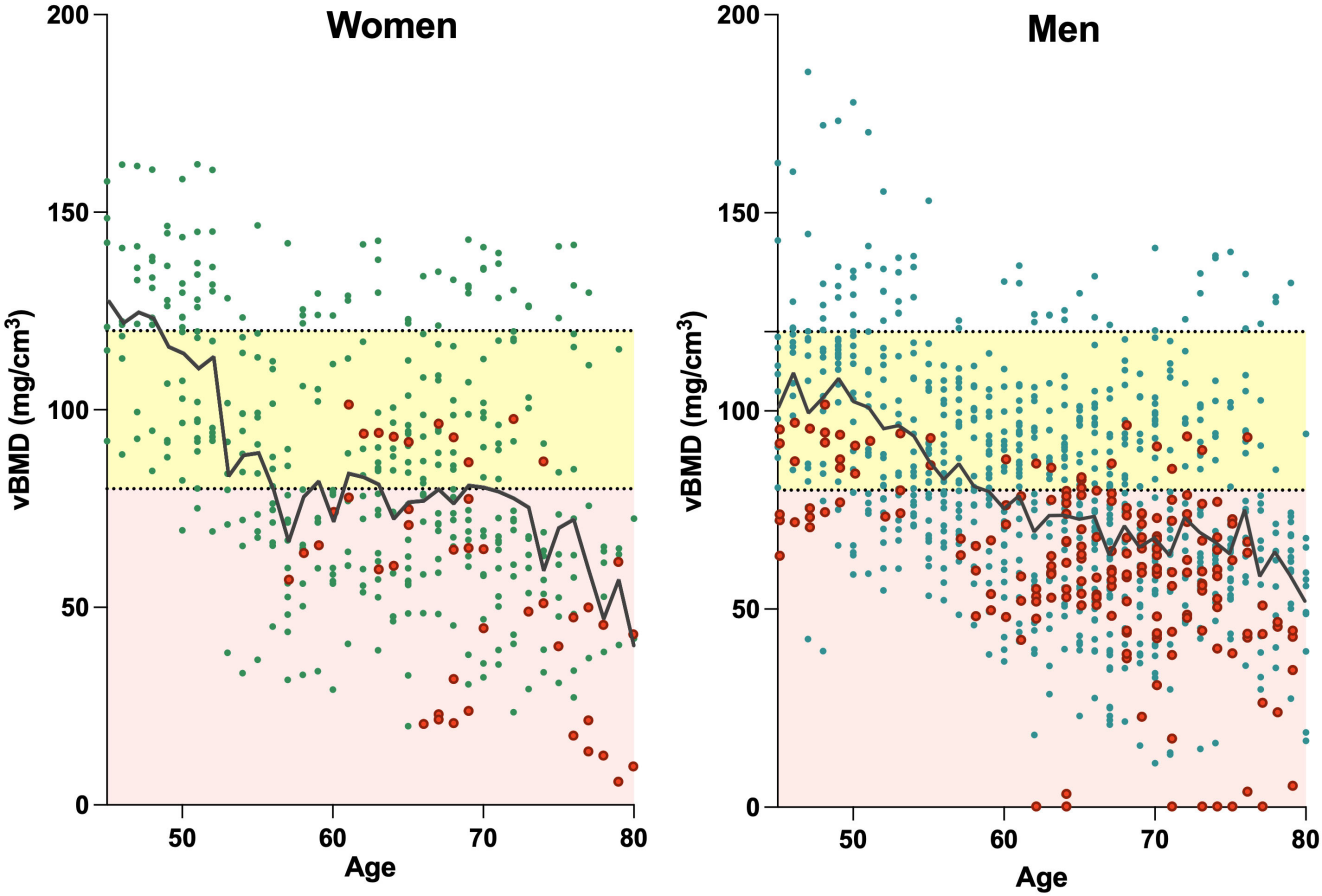
Relationship between age and vBMD for the lumbar spine (averaged values of L1-3 or L4 and L5) in women (left) and men (right). Data points include all available measurements of the patients over time, with red data points depicting individuals with VFs. Dotted horizontal lines denote the designated QCT cut-off values for osteopenia (<120 mg/cm^3^) and osteoporosis (<80 mg/cm^3^), while the areas within the respective ranges are highlighted in yellow (for osteopenia) and light red (for osteoporosis). The black lines illustrate the mean vBMD. Linear regression for women: y=-1.63*x+188.0; and men: y=-1.38*x+165.6. VF, vertebral fracture; QCT, quantitative computed tomography; vBMD, volumetric bone mineral density.

A total of 38 of the 168 included patients had pre-existing VFs or sustained a VF during the follow-up period (Genant grades 1-3). All prevalent or incident VFs occured in the range for osteopenia (vBMD <120mg/cm^3^) or below the cutoff for osteoporosis (vBMD <80mg/cm^3^) (**Figure 2**).

### 3.2 Changes in vBMD

In both women and men, the lumbar vBMD decreased steadily over time. There was no significant difference observed in the age-related changes in averaged lumbar vBMD (L1-3) between women and men (p=0.14).

**Figure 3:**
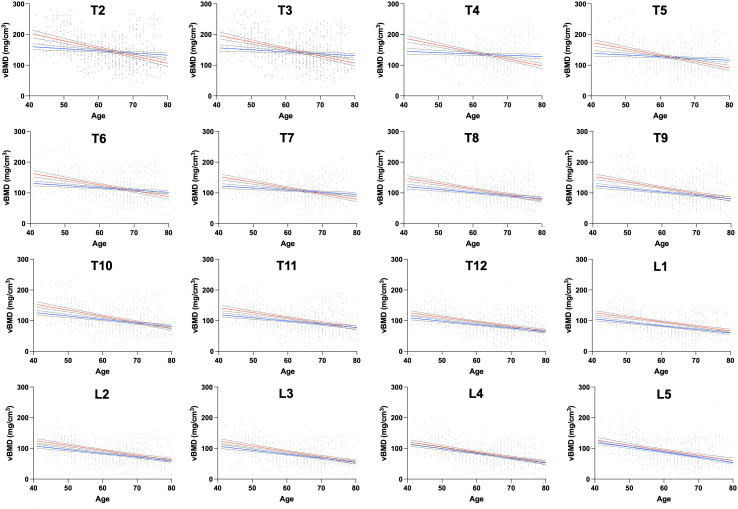
Relationship between level-wise thoracolumbar vBMD and age in a subgroup with pairs matched for sex- and age. T1 is not displayed to improve the readability of this figure. The straight lines (red=women; blue=men) represent linear regressions. The dashed lines represent the 95% CI. Statistically significant differences between the slopes were found at T1-11 levels. vBMD, volumetric bone mineral density; CI, confidence interval.

In the age- and sex-matched subgroup, single vertebral levels analysis revealed a trend toward a more pronounced vBMD decline in women at T12-L5, although it did not reach statistical significance. In contrast, at the thoracic spine (T1-T11), the slopes of vBMD decline differed significantly between women and men, with women exhibiting a more accelerated decline (p<0.01 to p<0.0001) (**Figure 3**).

### 3.3 Correlation of vBMD between the thoracic and the lumbar spine

**Figure 4:**
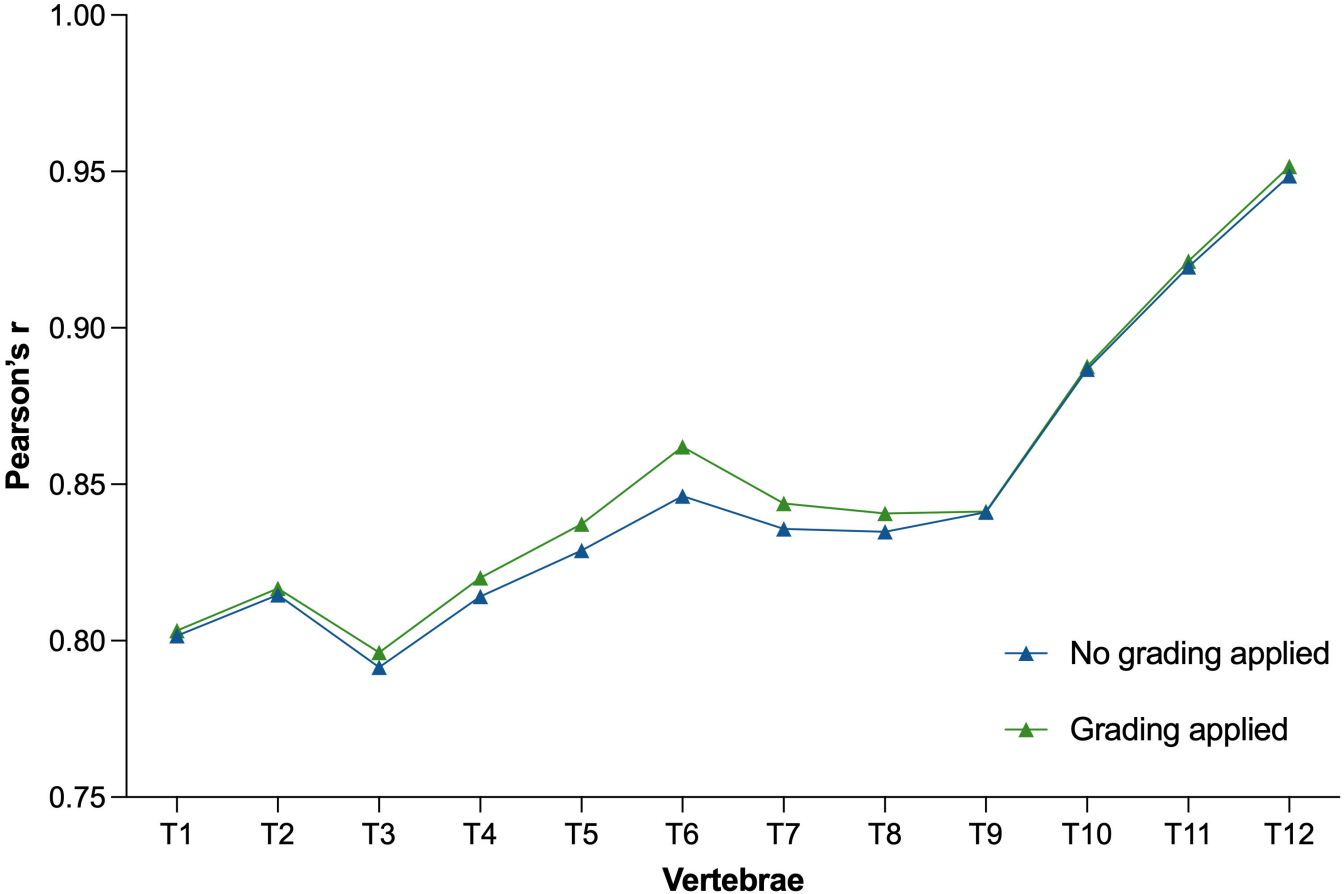
Plot showing Pearson’s correlation coefficients between vBMD T1 through T12 with respect to the averaged vBMD of L1–L3 before (blue) and after (green) exclusion of vertebrae due to fractures and degenerative changes. vBMD, volumetric bone mineral density.

The baseline vBMD at all thoracic levels strongly correlated with the averaged lumbar vBMD values at L1-L3, with a median Pearson’s correlation coefficient of r=0.85 (range: r T3 = 0.79 to r T12 = 0.95) (**Figure 4**). When not excluding fractured and degenerated vertebrae, the correlation slightly decreased to a median Pearson’s correlation coefficient value of r=0.84 (range: r T3 = 0.79 to r T12 = 0.94). The greatest decrease in correlation was observed at T3-T8 levels (not statistically significant).

### 3.4 Relationship between vertebral body volume and vBMD

**Figure 5:**
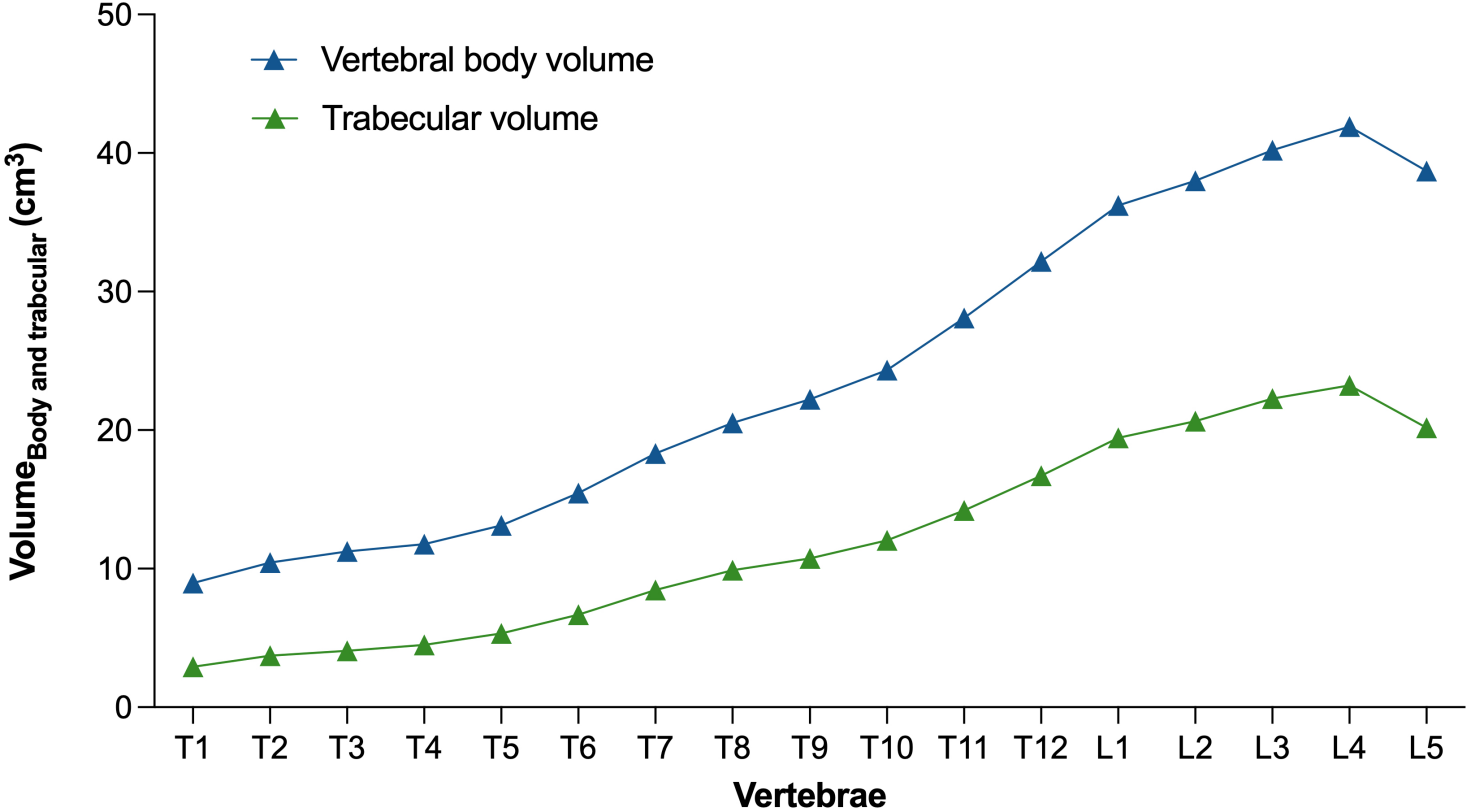
Level-wise vertebral body volume and trabecular volume at the thoracolumbar spine for both sexes. Data points represent the respective mean.

**Figure 6:**
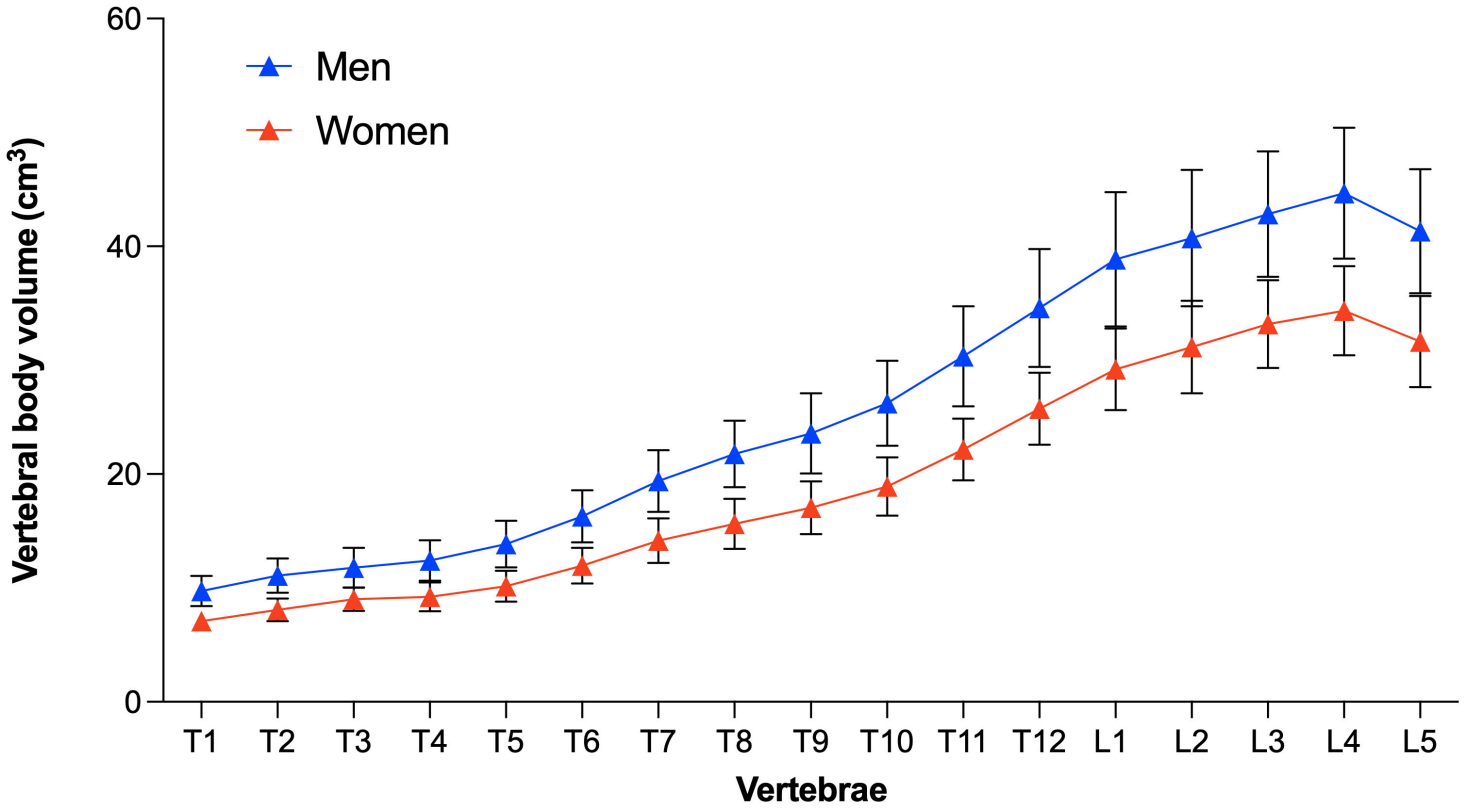
Level-wise vertebral body volume in women and men. Data points represent the respective mean and standard deviation.

**Figure 7:**
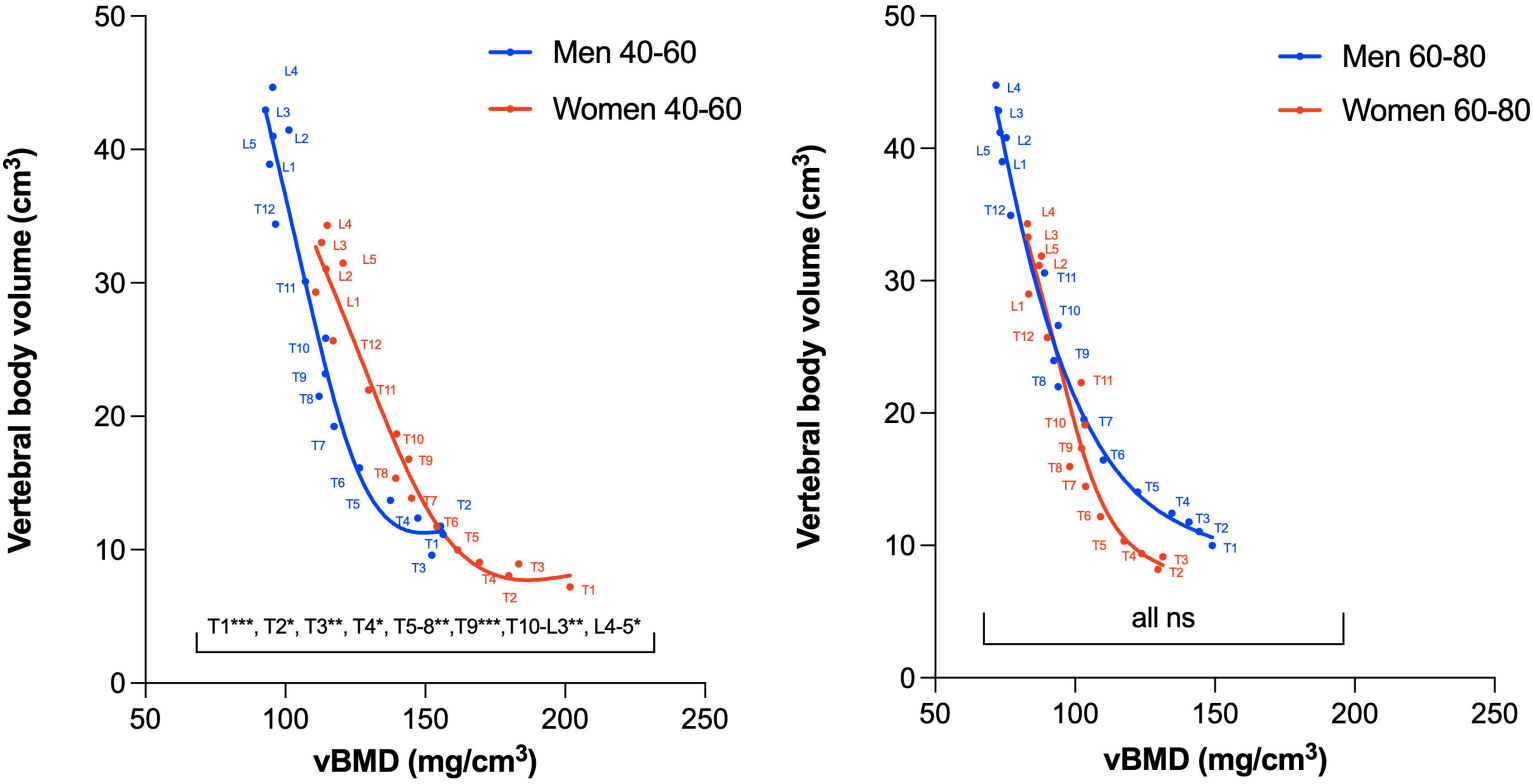
Association between vertebral body volume and vBMD in women and men aged 40-60 years (left) and women and men aged 60-80 years (right). In the 40-60 age group, the vBMD was significantly higher for all vertebral levels in women (red curve), whereas in the 60-80 age group, no significant difference was found for any vertebral level (due to a larger age-dependent vBMD decrease in women compared to men). Note, that the principal relationship between volume and density (i.e., the slope of the presented curves) all show similar trends independent of age groups and sexes. Due to insufficient data points, the T1 level is not shown for women in the 60-80 age group. vBMD, volumetric bone mineral density; ns, non-significant. * p<.05; **p<.01;***p<.001.

**Figures 5** and **6** show the distribution of the vertebral body volume and the trabecular volume at the thoracolumbar spine for both sexes, as well as the vertebral body volume for each sex separately. Both volumes increased gradually in the caudal direction until L4, with L5 having a slightly smaller body volume compared to L4. Across all vertebral levels, women tended to exhibit smaller vertebral volumes and bone mass than men (**Figure 6**, **Supplemantary Figure 2**). In contrast, these smaller vertebrae demonstrated higher vBMD at all levels in women aged 40-60 years compared to men (**Figure 7**). The differences in vBMD were significant for all vertebral levels (T1-L5) between women and men aged 40-60 years (p<0.05 to p<0.001). In the 60-80 years age group, all differences at T2-L5 levels were no longer significant. In both sexes and across age groups, there was a tendency for smaller vertebrae to exhibit higher vBMD and vice versa.

Single data points illustrating the relationship between vBMD and vertebral body volume in all patients at baseline are depicted in **Supplementary Figure 3**.

### 3.5 Longitudinal changes of vertebral body volume

**Figure 8:**
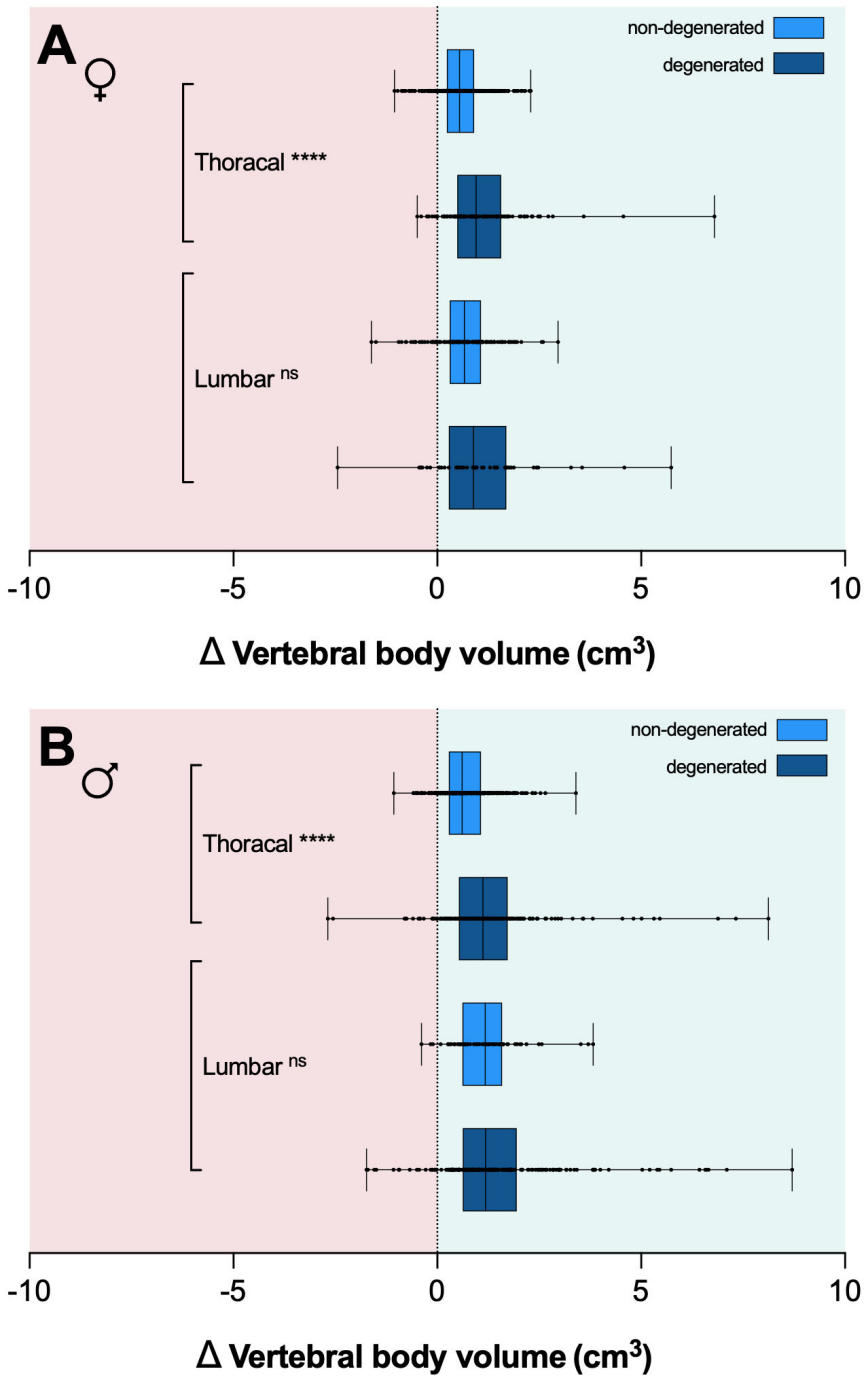
Box plots depicting the median in vertebral body volume change (Δ body volume in cm^3^) for women **(A)** and men **(B)** between baseline and last follow-up CT scans for degenerated (dark blue) and non-degenerated vertebrae (light blue). Error bars indicate the minimum and maximum. Exclusion of degenerative vertebrae resulted in a significant reduction of Δ volume change at the thoracic spine in both sexes. CT, computed tomography. **** p<.0001. ns, non-significant.

The longitudinal volumetric analysis (i.e., Δ volume change until the most recent examination) revealed a tendency towards an increase in vertebral body volume in both sexes (**Figure 8**). The observed volume increase at the thoracic spine was significantly smaller, after the exclusion of all degenerated vertebrae in women (mean volume increase_T1-12_ ± SD: 1.1 ± 1.0 cm^3^ vs. 0.6 ± 0.6 cm^3^) and men (mean volume increase_T1-12_ 1.2 ± 1.3 cm^3^ vs. 0.7 ± 0.6 cm^3^). A comparable trend, though not statistically significant, was evident in excluding degenerated vertebrae at the lumbar spine in women (mean volume increase _L1-5_ ± SD: 1.0 ± 1.4 cm^3^ vs. 0.7 ± 0.7 cm^3^) and men (mean volume increase _L1-5_ 1.5 ± 1.6 cm^3^ vs. 1.2 ± 0.8 cm^3^). The increase in volume between baseline and the last follow-up in non-degenerated thoracolumbar vertebrae was significantly different from zero for both sexes (p<0.0001). The mean change in volume (mean Δ volume change) in non-degenerated vertebrae was <5% of the respective vertebral body volumes.

## 4 Discussion

In the present study, we utilized opportunistic QCT and recent innovations in CNN-based frameworks to semi-automatically assess the 3D vertebral body volume and trabecular vBMD at the thoracolumbar spine (17). Using this pipeline, we replicated two anatomical findings. First, we demonstrated the well-established fact that women, on average, have smaller vertebrae compared to men, consistent with their smaller body size (4, 18). Second, our baseline data closely aligned with the absolute values and trend of increasing vertebral body volumes in the caudal direction (except for L5) as observed in two other CT-based studies on the physiological variability of vertebral body volumes in women and men (mean thoracic vertebrae volume 15.0 vs. 16.7 cm^3^; mean lumbar vertebrae volume 35.0 vs. 36.8 cm^3^) (10) and separately for the lumbar spine in women (36.7 vs. 31.9 cm^3^) and men (46.9 vs. 41.7 cm^3^) (9).

Our data also revealed more clinically relevant findings. When longitudinally examining VF status and vBMD, we exclusively identified prevalent and incident osteoporotic fractures falling within the lower range for osteopenia or below the threshold for osteoporosis. This may indirectly support the vBMD thresholds proposed by the American College of Radiology within our study cohort (16). When it comes to vBMD thresholds, another compelling idea is to utilize thoracic vBMD measurements for osteoporosis screening, similar to the already established threshold values for the lumbar spine (19). However, given the variations in vBMD at different spine regions, optimal thoracic thresholds are yet to be determined. In our study population, Pearson’s correlation coefficient indicated a strong relationship, probably explaining a large part of the variability in thoracic vBMD trough the variations in averaged vBMD at L1-3. This relationship could potentially be utilized to establish cut-off values for other parts of the spine, as already proposed for thoracic BMD as derived from coronary artery calcium scans (20).

When examining sex-specific differences in vBMD, numerous studies have reported that women exhibit higher lumbar vBMD, particularly before menopause (5–7). In a population-based study, this pattern was observed for L1-L3 vertebrae, using single-slice QCT-based measurements (5). In pairs of women and men from the Framingham study, men had about 9% lower trabecular vBMD at L3 (7). In a study using dual energy x-ray absorptiometry (DXA)-based measurements in pairs of opposite-sex twins, significantly higher vBMD was found in pre-menopausal females at L3, while no significant differences were found in the post-menopausal group (6). A more recent study has indirectly replicated these findings at L1 in a larger cohort, indicating that pre-menopausal women, on average, had higher L1 trabecular HU values compared with men, again showing similar values in both sexes after menopause (8).

We found these sex-specific differences to encompass all levels of the thoracolumbar spine, noting again that these significant differences were observed exclusively within the younger age group of 40-60 years. Women thereby appear to lose the potentially protective “advantage” of higher vBMD after menopause. Our results, indicating a significantly accelerated decline in vBMD at the thoracic spine in women, coupled with a similar (though in our cohort not significant) trend at the lumbar spine, suggest the mechanism potentially underlying these age-related differences. These observations align well with investigations in the Framingham study cohort, where thoracolumbar vBMD declined more rapidly for women than men (levels investigated: T8-10 and L3-5), and with a QCT-based study where vBMD declined faster in women at the lumbar spine (5, 21). In a prospective observational study investigating QCT-based lumbar vBMD in women during the menopausal transition, an accelerated rate of trabecular bone loss was also observed (22). Notably, when examining vBMD changes at the thoracic spine in men, we observed that not only do women experience a more accelerated decline in vBMD, but vBMD in men appeared almost preserved at the upper thoracic spine. A comparable phenomenon in bone density was also observed at the thoracic levels investigated in the aforementioned Framingham study (5).

Under identical loads, smaller bones, even when showing a similar vBMD, are inherently weaker than larger bones (4). In younger women, who are generally shorter and lighter than men on average, thus carrying similar relative loads compared to men, the observed higher vBMD at thoracolumbar levels may confer appropriate strength. However, given that bone size does not undergo the same changes as vBMD with age, this may represent an additional predisposition of elderly women to a significantly higher fracture risk after menopause.

When measuring age-related volumetric changes, bony degenerations, such as osteophytes, can render measurements heavily inaccurate. Through the exclusion of all degenerated vertebrae via semiquantitative assessment, our aim was to correct for such inaccuracies. The residual effect indicated a slight increase in vertebral volume over time in both sexes; however, these changes were subtle in comparison to the overall vertebral body volume. Moreover, the manual filtering step may not have been sensitive enough to exclude small or early degenerations, and slight over-segmentation in such cases could introduce a bias that must be considered during result interpretation. A potential solution could involve the use of an erosion algorithm to fully eliminate all bony extensions.

Post-mortem and CT-based studies have indicated an age-related increase in the cross-sectional area in men, whilst this phenomenon was absent in women (4, 21, 23–25). This difference is likely attributed to more pronounced periosteal apposition in men (18). In our study, we also observed a trend towards a more substantial increase in vertebral volume among men; however, both sexes exhibited a total volume increase throughout the thoracolumbar spine with age. Since we cannot exclude the possibility that minor bony degenerations contribute to this trend, it unfortunately remains uncertain which portion of the volume increase is attributable to periosteal apposition and what might potentially be a result of subtle osteophyte growth. In summary, we interpret our data to suggest that overall volume changes in the core vertebral body are only subtly apparent and more pronounced in men. A similar QCT-based study has also found only modest increase in the anterior column (i.e., vertebral body) volume in men, without significant changes in women (9). Correspondingly, our data also indicate that the age-related height loss at the spine is not primarily attributable to changes in vertebral body volume.

Several limitations should be acknowledged in interpreting the findings of this study. The relatively modest group size of 168 patients and the retrospective study design without a dedicated homogeneous imaging protocol may limit the generalizability of the results. Another consideration involves the use of a CNN-based framework to assess 3D volume, indicating the need for future studies to employ this methodology for validation purposes. The absolute volumetric values, naturally, depend on the study sample, especially in smaller samples. Nevertheless, our absolute volumetric values, and indirectly the precision of this method, closely aligned with findings observed in other CT-based studies (9, 10). Finally, the uneven distribution of data across different age groups, sexes, and vertebral levels further underscores the need for caution in drawing universal conclusions.

## 5 Conclusion

This study used opportunistic QCT and deep learning to investigate age- and sex-related changes in vBMD and vertebral body volume at the thoracolumbar spine. Knowing the physiological changes in vertebral body volume and vBMD might help in understanding the heterogeneous mechanisms that underlie individual VF risk.

## Data availability statement

The raw data supporting the conclusions of this article will be made available by the authors, without undue reservation.

## Ethics statement

The studies involving humans were approved by Ethikkommission der Fakultät für Medizin der Technischen Universität München. The studies were conducted in accordance with the local legislation and institutional requirements. Written informed consent for participation was not required from the participants or the participants’ legal guardians/next of kin in accordance with the national legislation and institutional requirements.

## Author contributions

SR: Conceptualization, Data curation, Formal analysis, Investigation, Methodology, Visualization, Writing – original draft, Writing – review & editing. JD: Conceptualization, Data curation, Investigation, Writing – original draft, Writing – review & editing. TM: Data curation, Investigation, Writing – review & editing. MH: Software, Writing – review & editing. JB: Conceptualization, Writing – review & editing. MH: Conceptualization, Writing – review & editing. ML: Conceptualization, Writing – review & editing. NS: Supervision, Writing – review & editing. TB: Supervision, Writing – review & editing. VS: Supervision, Writing – review & editing. MW: Supervision, Writing – review & editing. CZ: Funding acquisition, Supervision, Writing – review & editing. JK: Conceptualization, Formal Analysis, Funding acquisition, Investigation, Methodology, Project administration, Software, Supervision, Writing – review & editing.
