# Supplementary material for: Sex differences and age-related changes in vertebral body volume and volumetric bone mineral density at the thoracolumbar spine using opportunistic QCT
Source: Front Endocrinol (Lausanne). 2024 Feb 15;15:1352048. doi: 10.3389/fendo.2024.1352048 (PMC10911120; doi:10.3389/fendo.2024.1352048)
Supplement: Supplementary file 1 [file DataSheet_1.docx]

***Supplementary Material***

**Sex differences and age-related changes in vertebral body volume and volumetric bone mineral density at the thoracolumbar spine using opportunistic QCT**

Sebastian Rühling – Jonas Dittmann – Tobias Müller – Malek El Husseini – Jannis Bodden – Moritz R. Hernandez Petzsche – Maximilian T. Löffler – Nico Sollmann – Thomas Baum – Vanadin Seifert-Klauss – Maria Wostrack – Claus Zimmer – Jan S. Kirschke^*^

***Correspondence: jan.kirschke@tum.de**

**1 Supplementary Figures**

**
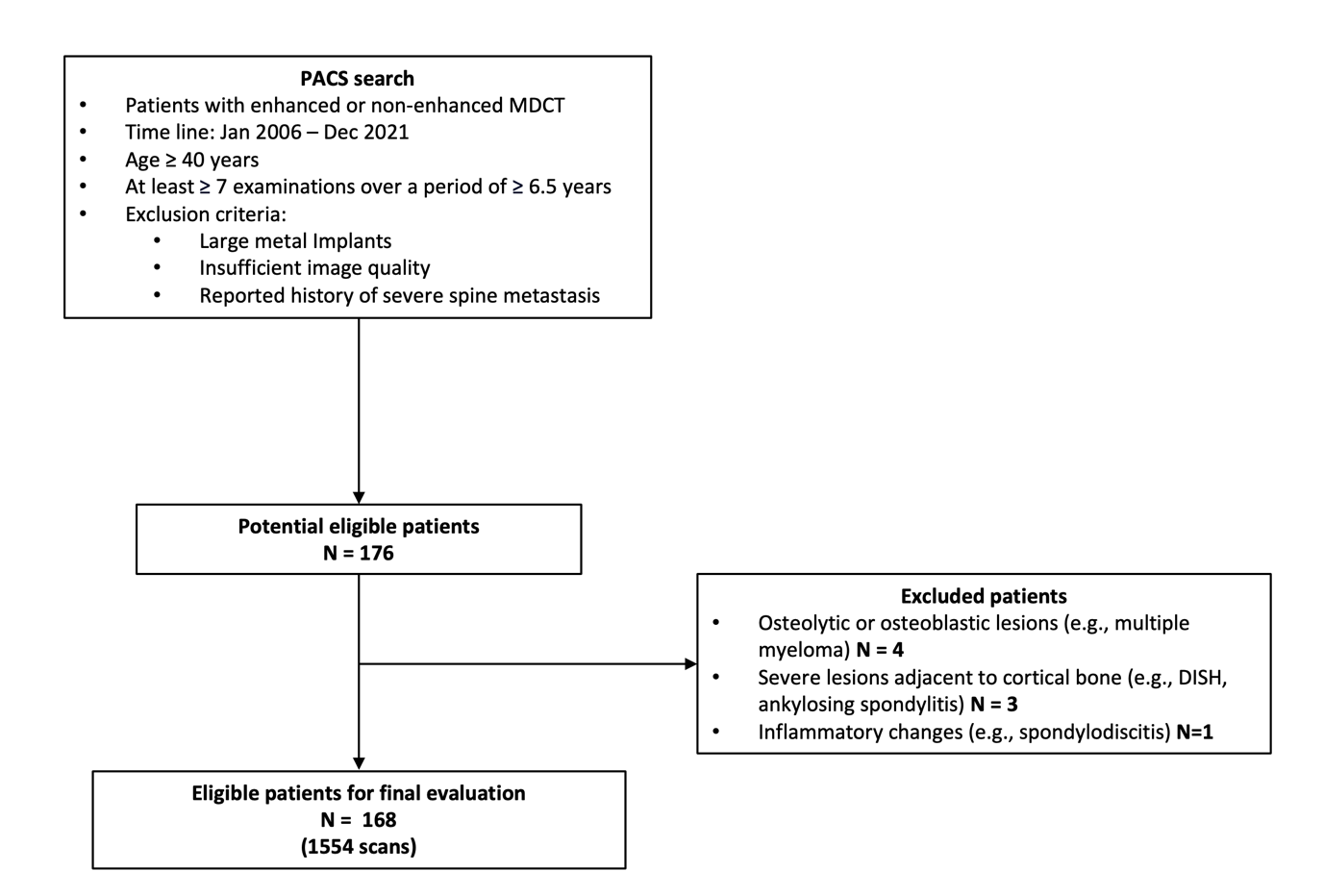
**

**Supplementary Fig. 1:** Flowchart depicting the data collection. MDCT = multidetector computed tomography; DISH = diffuse idiopathic skeletal hyperostosis.

**Supplementary Fig. 2:** Level-wise trabecular bone mass at the thoracolumbar spine in women and men aged 40-60 years (left) and women and men aged 60-80 years (right). Due to insufficient data points, the T1 level is not shown for women in the age group of 60-80 years.

**Supplementary Fig. 3** Level-wise association between vertebral body volume and vBMD. Scatterplots show measurements for women (red) and men (blue). vBMD=volumetric bone mineral density.
